# miR-155 mediates drug resistance in osteosarcoma cells via inducing autophagy

**DOI:** 10.3892/etm.2014.1752

**Published:** 2014-06-02

**Authors:** LU CHEN, KE JIANG, HUA JIANG, PENG WEI

**Affiliations:** 1 Orthopedics Department, Affiliated Hospital of North Sichuan Medical College, Nanchong, Sichuan 637000, P.R. China; 2 Orthopedics Department, Second People’s Hospital of Chengdu, Chengdu, Sichuan 610017, P.R. China

**Keywords:** microRNA-155, drug resistance, osteosarcoma cells, autophagy

## Abstract

Frequent acquisition of drug resistance is often associated with the chemotherapy of malignant tumors, including osteosarcoma. A number of studies have demonstrated a critical role for autophagy in osteosarcoma development, therapy and drug resistance. However, the molecular mechanisms underlying the autophagy-mediated chemotherapy resistance of osteosarcoma cells remain largely unknown. In the present study, we determined the autophagy and microRNA-155 (miR-155) expression induced by chemotherapeutic drugs in osteosarcoma cells. Then we determined the promotory role of miR-155 to the chemotherapy-induced autophagy. Our results demonstrated that microRNA-155 (miR-155) expression was highly induced during chemotherapy of osteosarcoma cells, and this was accompanied by upregulated autophagy. The increased miR-155 expression levels upregulated anticancer drug-induced autophagy in osteosarcoma cells and ameliorated the anticancer drug-induced cell proliferation and viability decrease. Therefore, the results of the present study demonstrated that miR-155 mediated drug-resistance in osteosarcoma cells by inducing autophagy. The present study recognized a novel mechanism of chemoresistance in osteosarcoma cancers.

## Introduction

Osteosarcoma is the eighth most common type of cancer found in children and adolescents, accounting for 2.4% of all malignancies in pediatric patients and ~20% of all primary bone cancers (1). Chemotherapy is the first choice treatment for osteosarcoma, with multiple anticancer drugs, including doxorubicin, cisplatin and high-dose methotrexate (2,3). In the last three decades, neoadjuvant chemotherapy has increased the long-term survival rate of osteosarcoma patients from <20 to ~80% (4–6). However, patients that are less responsive to these drugs have a poor prognosis. In addition, the frequent acquisition of drug resistance and the occurrence of ‘secondary malignancies’ are often associated with chemotherapy and are significant obstacles to achieving favorable outcomes. Thus, it is important to identify the molecular mechanisms underlying the drug resistance of osteosarcoma cancer cells.

Drug resistance of osteosarcoma cancer cells has been attributed to various mechanisms, including dysfunctional membrane transport (7), resistance to apoptosis (8), persistence of stem cell-like tumor cells (9) or autophagy (7). In addition, osteosarcoma tumors have been reported to exhibit a multidrug resistance phenotype (10). Autophagy is a lysosomal degradation pathway that is essential for cell growth, survival, differentiation, development and homeostasis (11). It is a tightly regulated process that helps to maintain a balance among the synthesis, degradation and subsequent recycling of cellular products. A number of studies have demonstrated a critical role for autophagy in cancer development and therapy (9,12,13). Previous studies hypothesized that autophagy is facilitated following treatment with cytotoxic agents (14–17). However, the mechanisms underlying autophagic drug resistance in osteosarcoma therapy remain largely unknown.

MicroRNAs (miRNAs) are family of endogenous non-coding RNA molecules that are comprised of 22 nucleotides, which regulate gene expression (18) in organisms ranging between nematodes and humans and in a broad array of mammalian cell processes (19). Recently, miRNAs have been associated with cell chemosensitivity or chemotherapy resistance in a variety of cancer cell types (20–24), including osteosarcoma (25). miR-140 was reported to be involved in the chemoresistance of osteosarcoma cells via the suppression of histone deacetylase 4, which in turn reduced cell proliferation (25). Furthermore, an increasing number of studies have demonstrated that miRNA molecules regulate cellular autophagy processes (26–28). Zhu *et al* (27) reported that miR-30a targets beclin 1, resulting in decreased autophagic activity. In addition, Brest *et al* (28) showed that a miR-196-based alteration in the expression of immunity-related GTPase family M protein can affect the efficacy of autophagy. However, the role of miRNAs in autophagy-mediated chemotherapy resistance in osteosarcoma remains unknown.

The aim of the present study was to investigate the expression levels of miR-155 in osteosarcoma cells following chemotherapy and analyze the association with chemotherapy resistance *in vitro*. The effect that miR-155 overexpression exhibited on autophagy was also investigated with the aim of demonstrating a novel role for miR-155 in chemotherapy resistance during the treatment of osteosarcoma.

## Materials and methods

### Cell culture and reagents

Human osteosarcoma cell lines (Saos-2 and MG-63) were obtained from the Cell Resource Center of the Chinese Academy of Medical Sciences (Beijing, China). The cells were cultured in Eagle’s Minimum Essential Medium (Invitrogen, Carlsbad, CA, USA) or McCoy’s 5A Modified Medium (Invitrogen) supplemented with 10% fetal bovine serum (GIBCO, Rockville, MD, USA), and were incubated at 37°C with 5% CO_2_. Antibodies against GAPDH, LC3-II and autophagy protein 5 (Atg5) were obtained from Santa Cruz Biotechnology, Inc. (Santa Cruz, CA, USA) and rapamycin was purchased from Sigma-Aldrich (St. Louis, MO, USA). The coding sequence of microtubule-associated protein 1-LC3 fusion with green fluorescent protein (GFP) was synthesized and cloned into pcDNA3.1(+) to construct the LC3-GFP-expressing plasmid.

### Autophagic vesicles quantified by GFP-LC3

Quantitative GFP-LC3 light microscopy autophagy assays were performed in Saos-2 cells with various treatments. Cells were grown to 80% confluency and were transfected with a GFP-LC3-expressing plasmid using Lipofectamine 2000 (Invitrogen Life Technologies, Carlsbad, CA, USA). At 24 h following transfection, the cells were subjected to 50 nM rapamycin (Sigma-Aldrich), 0.2 μg/ml doxorubicin (Dox; Sigma-Aldrich) or 20 μM cisplatin (Cis; Sigma-Aldrich) for an additional 24 h. In a separate experiment, cells were simultaneously and additionally transfected with 20 nM miR-155 and analyzed with fluorescence microscopy. The number of punctate GFP-LC3 dots in each cell was counted and at least 100 cells were included for each group.

### miRNA extraction and quantitative polymerase chain reaction (qPCR)

Total miRNA extraction was performed using a mirVana miRNA Isolation kit (Ambion, Inc., Austin, TX, USA). Quantification of miR-155 expression was conducted using the mirVana qRT-PCR miRNA Detection kit (Ambion, Inc.), where U6 small nuclear RNA was used as an internal control. The ^ΔΔ^Ct method was used for relative quantification (29). A non-radioactive northern blot method (LED) for small RNA (15–40 bases) detection, using digoxigenin-labeled oligonucleotide probes containing locked nucleic acids (Roche Diagnostics, GmbH, Mannheim, Germany) and 1-ethyl-3-(3-dimethylaminopropyl) carbodiimide, (Sigma-Aldrich was utilized to confirm the miR-155 and U6 expression levels, according to the protocol previously described (30).

### Western blot analysis

Cell extracts were prepared according to the standard protocol, and protein expression levels were detected by western blot analysis using polyclonal (rabbit) anti-LC3-II, anti-Atg5 or anti-GAPDH antibodies. Goat anti-mouse IgG or goat anti-rabbit IgG (Pierce Biotechnology, Inc., Rockford, IL, USA) secondary antibodies, that were conjugated to horseradish peroxidase, were used for detection via an enhanced chemiluminescence detection system (Super Signal West Femto, Pierce Biotechnology, Inc.).

### Cell proliferation assay

Cell viability was expressed as the relative percentage of viable cells to control human umbilical vein endothelial cells. For the proliferation assay, following transfection with miR-155 mimics or miR-155 control, cells were incubated with Cell Counting Kit-8 (CCK-8; Dojindo Molecular Technologies, Inc., Kumamoto, Japan). The absorbance of each well at 450 nm was detected following visual color occurrence at 24, 48 or 72 h. Independent experiments were performed in triplicate.

### Statistical analysis

For GFP-LC3 dot number analysis, relative miR-155 expression, conversion of LC3-I to LC3-II, relative expression of Atg5 against GAPDH and CCK-8 measurements, the statistical evaluations are presented as the mean ± SE. Data were analyzed using the Student’s t test. All data were analyzed by the SPSS v16.0 (SPSS Inc., Chicago, IL, USA). P<0.05 was considered to indicate a statistically significant result.

## Results

### Autophagy is induced during chemotherapy in osteosarcoma cells

**Figure 1:**
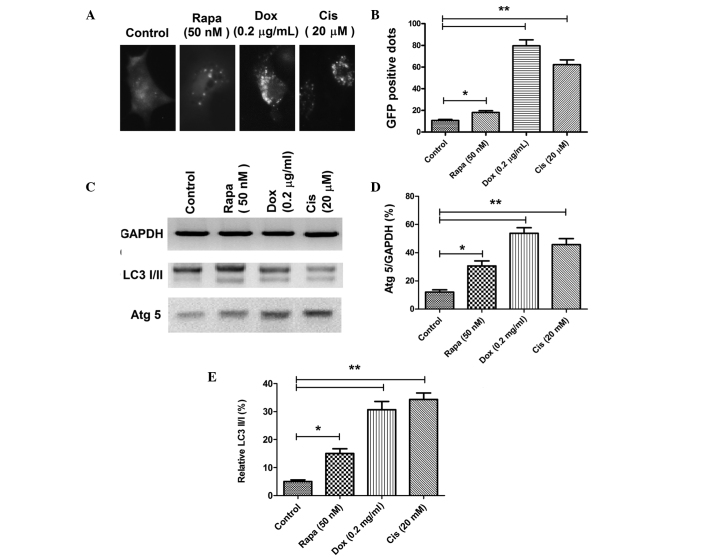
Autophagy was induced during chemotherapy in osteosarcoma cells. (A) LC3 punctas in Saos-2 cells following chemotherapy. (B) Quantitative analysis of the punctate GFP-LC3 dots in Dox- or Cis-treated Saos-2 cells. (C) Western blotting of Atg 5 and LC3-I/II in Dox- or Cis-treated Saos-2 cells; (D) Relative expression of Atg 5 to GAPDH in Dox- or Cis-treated Saos-2 cells; (E) Relative expression of LC3-II to LC3-I in Dox- or Cis-treated Saos-2 cells.. Data were normally distributed. The difference between two groups in the GFP-positive dots, the relative expression of Atg 5 and LC3-II/I was analyzed using the Student-Newman-Keuls test. Independent experiments were performed in triplicate. ^*^P<0.05 and ^**^P<0.01, vs. control. GFP, green fluorescent protein; Dox, doxorubicin; Cis, cisplatin; Atg5, autophagy protein 5.

An increasing number of studies have indicated that autophagy is induced in cancer cells following treatment with cytotoxic agents (14–17). To confirm the autophagy level in osteosarcoma cells following Dox or Cis treatment, autophagic acidic vesicular organelles and LC3 punctas (31) were detected via a GFP-LC3 report vector and fluorescence microscopy in the Saos-2 osteosarcoma cell line. The conversion between LC3-I and LC3-II, as well as Atg5 expression, one of the autophagy-related gene products, was then quantified by western blot analysis. Accumulation of LC3 punctas in osteosarcoma cells was significantly higher in the Dox (0.2 μg/ml) or Cis (20 μM) treatment groups (Fig. 1A); there were more GFP-positive dots (autophagic vesicles) in the Dox- or Cis-treated Saos-2 cells when compared with the control Saos-2 cells (P<0.05; Fig. 1B). In addition, immunoblot analysis revealed significantly higher conversion levels of LC3-I to LC3-II and a higher level of Atg5 expression in the osteosarcoma cells that had been treated with 0.2 μg/ml Dox or 20 μM Cis (Fig. 1C–E). These observations indicated that chemotherapy in osteosarcoma cells induces autophagy.

### miR-155 expression increases in osteosarcoma cells following treatment with chemotherapy agents

**Figure 2:**
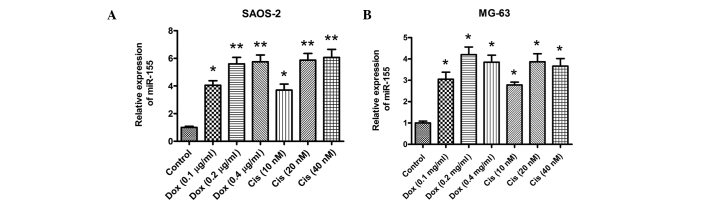
miR-155 expression was upregulated in osteosarcoma cells following treatment with chemotherapeutic drugs. qPCR analysis showing the relative miR-155 expression to U6 in (A) Saos-2 and (B) MG-63 cells. All the experiments were performed in triplicate. ^*^P<0.05 or ^**^P<0.01, vs. control. miR-155, microRNA-155; qPCR, quantitative polymerase chain reaction.

The role of miRNAs in chemotherapy-induced autophagy of cancer cells remains unknown. To screen possible miRNAs that may be important for anticancer drug-induced autophagy in osteosarcoma cells, miRNA expression levels were analyzed by microarray in osteosarcoma cells following treatment with Dox (data not shown). miR-155 was demonstrated to be the most highly-expressed miRNA. Thus, the expression level of miR-155 was quantified in Saos-2 and MG-63 cells following treatment with Dox or Cis. The results indicated that treatment with 0.2 μg/ml Dox or 20 μM Cis significantly upregulated the miR-155 expression levels in the two cell lines. A qPCR assay demonstrated that significantly higher expression levels of miR-155 were induced in Saos-2 or MG-63 cells following Dox or Cis treatment (Fig. 2A and B). Therefore, miR-155 expression is induced *in vitro* during anticancer drug therapy in osteosarcoma cells.

### Overexpression of miR-155 ameliorates the anticancer drug-induced cell proliferation decrease

**Figure 3:**
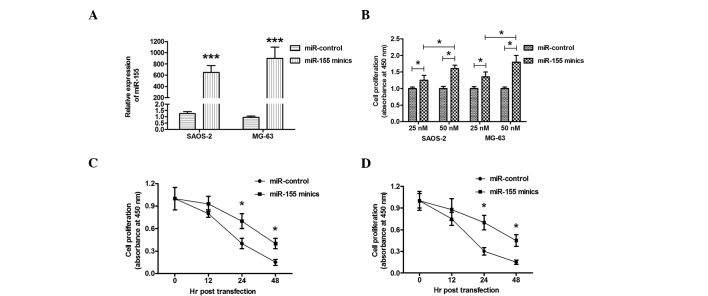
Overexpression of miR-155 ameliorated the anticancer drug-induced cell proliferation decrease *in vitro*. (A) Expression of miR-155 was compared following transfection with miR-control or miR-155 mimics (25 nM) in Saos-2 or MG-63 cells. (B) Cell proliferation of Saos-2 or MG-63 cells following miR-155 mimics or miR-control transfection at 25 nM or 50 nM, as determined by a CCK-8 assay. Growth curves showing the cell proliferation following 0.2 μg/ml Dox treatment and miR-155 mimics or miR-control transfecion in (C) Saos-2 and (D) MG-63 cells, as determined by a CCK-8 assay. Independent experiments were performed in triplicate. ^*^P<0.05, ^**^P<0.01 and ^***^P<0.001, vs. control miR-155, microRNA-155; CCK-8, Cell Counting Kit-8; Dox doxorubicin.

To determine the possible effect of miR-155 on osteosarcoma cell proliferation, the proliferation of Saos-2 or MG-63 cells that had been treated with Dox and transfected with miR-155 mimics was determined using a CCK-8 assay. Transfection with miR-155 mimics significantly upregulated the miR-155 level in Saos-2 or MG-63 cells (Fig. 3A). The elevated miR-155 expression in the Saos-2 or MG-63 cell lines ameliorated the cell proliferation retardation that had been caused by Dox in a dose-dependent manner, when compared with the miRNA control (Fig. 3B). To further observe the effect of miR-155 overexpression on cell proliferation, the proliferation of Saos-2 or MG-63 cells was determined at various time points following miR-155 mimics transfection. As shown in Fig. 3C and D, miR-155 mimics transfection resulted in a time-dependent amelioration of the cell proliferation decrease in Saos-2 and MG-63 cells. Thus, overexpression of miR-155 ameliorated the anticancer drug-induced cell proliferation decrease in osteosarcoma cells.

### Overexpression of miR-155 upregulates anticancer drug-induced autophagy in osteosarcoma cells

**Figure 4:**
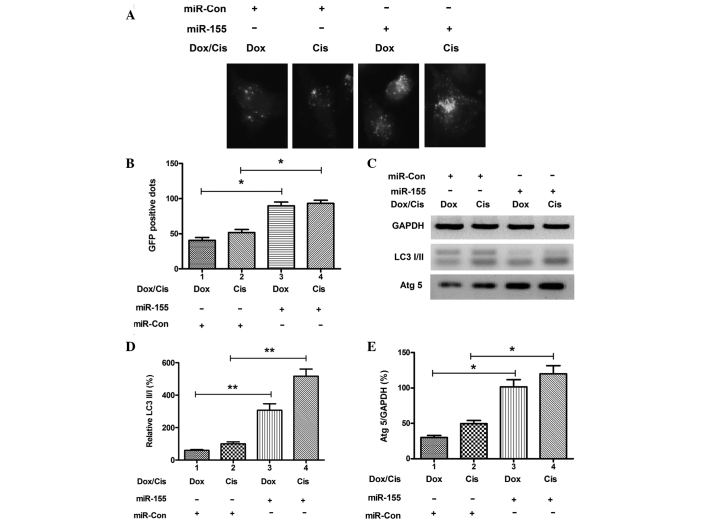
Overexpression of miR-155 upregulated anticancer drug-induced autophagy in osteosarcoma cells. (A) LC3 punctas under fluorescence microscopy in Saos-2 cells following 0.05 μg/ml Dox or 5 μM Cis treatment and miR-155 mimics or miR-control transfection. (B) Quantitative analysis of the punctate GFP-LC3 dots. (C) Western blotting of LC3-I/II and Atg 5 in miR-155 mimics-transfected Saos-2 cells; (D) Relative expression of LC3-II to LC3-I in miR-155 mimics-transfected Saos-2 cells; (E) Relative expression of Atg 5 to GAPDH in miR-155 mimics-transfected Saos-2 cells. Independent experiments were performed in triplicate. ^*^P<0.05 or ^**^P<0.01, vs. control. miR-155, microRNA-155; Dox, doxorubicin; Cis, cisplatin; GFP, green fluorescent protein; Atg5, autophagy protein 5.

To determine the possible contribution of miR-155 to autophagy in drug-treated osteosarcoma cells, miR-155 expression was manipulated in Saos-2 cells via transfection with miR-155 mimics or miRNA control. Transfection with miR-155 mimics significantly upregulated the miR-155 expression level in the cells (Fig. 3A; P<0.001). The level of autophagy was determined in Saos-2 cells following miR-155 mimics transfection. As shown in Fig. 4A and B, there were more GFP-positive dots (LC3 punctas) in the Saos-2 cells that had been transfected with miR-155 mimics when compared with transfection with miRNA control (P<0.05). In addition, significantly higher conversion levels of LC3-I to LC3-II and high expression levels of Atg5 were also confirmed in the osteosarcoma cells transfected with miR-155 mimics (P<0.01 and P<0.05 respectively; Fig. 4C–E). These results confirm that overexpression of miR-155 contributes to anticancer drug-induced autophagy in osteosarcoma cells.

## Discussions

Chemoresistance to anticancer therapeutic drugs is a common occurrence and contributes to cancer mortality, as it often leads to failure in the blockage of disease progression. A number of studies have evaluated the mechanisms of resistance and the biological factors involved (32). Autophagy is an intracellular self-protective mechanism that prevents the toxic accumulation of damaged components, but also recycles the degraded components to sustain metabolic homeostasis. Upregulated autophagy has been found in a wide variety of cancer cells faced with metabolic and therapeutic stress, and has been shown to contribute to the chemotherapy resistance of various types of tumors (4,33). Blocking cancer cell autophagy is emerging as a novel approach to enhance the efficiency of chemotherapy in cancer treatment (8,9). The results of the present study demonstrated that treatment with Dox or Cis caused the activation of autophagy in osteosarcoma cells, which enhanced autophagy and facilitated the survival of tumor cells under these drug treatments. The results from the *in vitro* experiments demonstrated that miR-155 expression was also enhanced in osteosarcoma cells following anticancer treatment, and the upregulated miR-155 expression mediated the autophagy.

Tight control of autophagy is essential for normal or tumor cells to survive, and recent advances in this field have begun to unveil the molecular mechanisms underlying autophagy regulation (34). Within the past decade, genetic screening in yeast has identified a large family of core autophagy regulators, the Atg-related genes, a number of which have known orthologs in mammalian cells that serve to coordinately regulate the stepwise progression of this degradation pathway (35,36). In addition, a diverse and complex network of upstream signaling pathways contribute to autophagy regulation, including the phosphatidylinositol 3-kinase, RAS and AMP-activated protein kinase pathways, a number of which converge at mammalian target of rapamycin complex 1, a key negative regulator of autophagy signaling (37,38). There is much evidence that miRNA molecules are differentially expressed in human cancers, in which they function as tumor suppressors or oncogenes, with their oncogenic regulation spanning from initiation, progression to metastasis and treatment sensitivity. Notably, miRNAs can regulate a multitude of targets and biological networks in autophagy (39,40). A previous study indicated clear roles of miRNAs in autophagy induction, autophagic vesicle nucleation, autophagic vesicle elongation and vesicle fusion to lysosomes (39). The present study confirmed that during treatment with Dox or Cis in osteosarcoma cells, miR-155 expression was strongly induced. The increased miR-155 expression facilitated tumor cell proliferation via upregulating autophagy, thus, facilitated the resistance of osteosarcoma cells to Dox or Cis. However, the details underlying the mechanism of miR-155-mediated autophagic resistance in osteosarcoma cells requires further study.

In conclusion, the present study has demonstrated that anticancer drug treatment upregulates miR-155 expression in osteosarcoma cells. Overexpression of miR-155 induces the activation of autophagy, which promotes tumor cell survival and chemoresistance. These observations reveal a novel role for miR-155 in chemotherapy resistance during the treatment of osteosarcoma.
